# Quantification of liver iron overload disease with laser ablation inductively coupled plasma mass spectrometry

**DOI:** 10.1186/s12880-018-0291-3

**Published:** 2018-12-04

**Authors:** Philipp Kim, Sabine Weiskirchen, Ricarda Uerlings, Astrid Kueppers, Florian Stellmacher, André Viveiros, Heinz Zoller, Ralf Weiskirchen

**Affiliations:** 1 Institute of Molecular Pathobiochemistry, Experimental Gene Therapy and Clinical Chemistry (IFMPEGKC), RWTH University Hospital Aachen, Pauwelsstr 30, D-52074 Aachen, Germany; 2 Central Institute for Engineering, Electronics and Analytics, ZEA-3, Forschungszentrum Jülich, Jülich, Germany; 3 Pathology, Research Center Borstel, Borstel, Germany; 4 Department of Internal Medicine II, Medical University of Innsbruck, Innsbruck, Austria

**Keywords:** Metal, Iron, Hemochromatosis, Mass spectrometry, Molecular imaging, Liver, Iron overload, *HFE*, Diagnostic, Mutation

## Abstract

**Background:**

Hereditary hemochromatosis is the most frequent, identified, genetic disorder in Caucasians affecting about 1 in 1000 people of Northern European ancestry, where the associated genetic defect (homozygosity for the p.Cys282Tyr polymorphism in the *HFE* gene) has a prevalence of approximately 1:200. The disorder is characterized by excess iron stores in the body. Due to the incomplete disease penetrance of disease-associated genotype, genetic testing and accurate quantification of hepatic iron content by histological grading of stainable iron, quantitative chemical determination of iron, or imaging procedures are important in the evaluation and staging of hereditary hemochromatosis.

**Methods:**

We here established novel laser ablation inductively coupled plasma mass spectrometry protocols for hepatic metal bio-imaging for diagnosis of iron overload.

**Results:**

We demonstrate that these protocols are a significant asset in the diagnosis of iron overload allowing iron measurements and simultaneous determination of various other metals and metalloids with high sensitivity, spatial resolution, and quantification ability.

**Conclusions:**

The simultaneous measurement of various metals and metalloids offers unique opportunities for deeper understanding of metal imbalances. Laser ablation inductively coupled plasma mass spectrometry (LA-ICP-MS) is a highly powerful and sensitive technique for the analysis of a variety of solid samples with high spatial resolution. We conclude that this method is an important add-on to routine diagnosis of iron overload and associated hepatic metal dysbalances resulting thereof.

**Electronic supplementary material:**

The online version of this article (10.1186/s12880-018-0291-3) contains supplementary material, which is available to authorized users.

## Background

Genetic iron storage diseases encompass a genetically heterogeneous group of disorders with strong environmental modifiers of disease expression [1]. Homozygozity for the c845G > A mutation within the *HFE* gene (OMIM: 613609) causing a substitution of the cysteine with a tyrosine residue in position 282 of the *HFE* gene product (p.Cys282Tyr) is, by far, the most frequent genetic form of iron overload. Autosomal recessive *HFE*-related iron overload is associated with typical clinical symptoms resulting from excess iron in several organs, mainly the liver representing the main storage site for iron. These include hepatomegaly, cirrhosis, hepatocellular carcinoma, diabetes mellitus, cardiomyopathy, hypogonadism, arthropathy, and increased skin pigmentation [2].

Genetic testing should be requested in patients with increased transferrin saturation, but results from population-based genetic screening studies have shown that the homozygosity for the p.Cys282Tyr polymorphism is associated with hemochromatosis in only 14% of individuals with this potentially disease-associated genotype. As hemochromatosis can also be caused by other mutation in the HFE gene or so called non-HFE hemochromatosis genes (*HJV*, *HAMP*, *TFR2*), homozygosity for p.Cys282Tyr is neither sufficient nor necessary for the diagnosis hemochromatosis. Accurate quantification of hepatic iron content by histological grading of stainable iron using Prussian blue or other dyes, direct chemical iron determination as well as magnetic resonance imaging (MRI) therefore remain essential diagnostic tools to detect and evaluate abnormal iron deposition in hereditary hemochromatosis (HH).

Genetic testing for common *HFE* variants associated with iron overload (C282Y, H63D) is a simple and economical procedure to confirm the diagnosis HH. A large variety of high-throughput methods for *HFE* genotyping are in daily routine use [3]. In addition, the repertoire of methods used for genetic diagnosis of HH contains other less commonly used methods such as PCR and reverse hybridization, direct sequencing, allele-specific PCR, PCR and high-resolution melting or single-stand conformational polymorphism, pyrosequencing and singe base extension [3]. Although classical liver biopsy with determination of hepatic iron concentration in fresh and paraffin-embedded tissue [4, 5] that has long been the gold standard for diagnosis of HH lost in importance, liver biopsy continues to have a very important diagnostic and prognostic implications in several hemochromatosis patients, especially in patients with suspected non-*HFE* hemochromatosis [6]. Such histochemical reactions are used to detect the presence of iron in biopsy specimens by forming insoluble complexes with appropriate dyes. Moreover, quantitative tissue iron determination and calculation of the hepatic iron index, defined as a quotient of micromoles or iron per gram of dry liver tissue and age of patient in years, was introduced to distinguish early hemochromatosis from alcoholic siderosis [7]. However, conventional tissue-ashing protocols, which rely on prolonged exposure to heat and caustic acids to achieve complete tissue decomposition are time-consuming, prone to sample loss and contamination, and potentially dangerous (e.g. release of hazardous fumes). Therefore, these procedures are for occupational medical reasons critical to adapt to routine clinical laboratories.

Most importantly, the subsequent determination of iron by atomic absorption spectrometry or chemically titration allows only the absolute quantitation of iron concentrations, but is not suitable to identify patterns, zonal gradients, or abnormal regional deposits or iron within the tissue. A histological hallmark of HH is that the accumulation and deposition or iron initially occurs in periportal hepatocytes (zone 1) in early phases of disease, while it extends to midzonal (zone 2) and centrilobular (zone 3) hepatocytes and biliary epithelium during progression of disease [8]. In contrast, transfusional iron overload, alcoholic siderosis or iron overload caused by SLC40A1 mutations are typically characterized by iron laden macrophages. Therefore, regional differences in hepatic iron concentrations are relevant providing information about the cause of iron overload, its stage and progression of the disease.

Recently, we established novel laser ablation inductively coupled plasma mass spectrometry (LA-ICP-MS)-based protocols for hepatic metal bio-imaging. Here we demonstrate that these protocols are a significant asset in the diagnosis of iron overload allowing iron measurements with high sensitivity, spatial resolution, and quantification ability. These protocols will be valuable in estimating liver iron content in all forms iron imbalances resulting from genetic disorders (hemochromatosis, β-thalassemia, and aceruloplasminemia), iron malabsorption, internal chronic bleeding, excessive menstrual bleeding, and nutritional deficiencies resulting for example from strict vegetarian food consumption.

## Methods

### Human samples

Percutaneous liver biopsy specimen analyzed in this study (*n* = 4 controls, n = 4 patients with confirmed iron overload) came from the Department of Medicine II (Gastroenterology and Hepatology) located at the University Innsbruck in Austria. All samples have been collected in the course of routine clinical care and were obtained by a standardized protocol. Ultrasound guided biopsy of the liver was carried out through an intercostal space in the right midclavicular line, where at least 5 cm liver parenchyma without large vessels were visible in expiratory hold. Before taking a biopsy with an 18-gauge × 4.5 in. Trucut® needle, skin disinfection and a sterile 4 mm skin incision was performed under local anesthesia with 5–10 ml 1% lidocaine injected subcutaneously and under the liver capsule. Clinical data relevant on iron metabolism and *HFE* genetics are summarized in Table 1. The depicted values for transferrin, ferritin, and transferrin saturation in this table were determined in a laboratory using standard laboratory test. The serum and liver tissue iron were determined by atomic absorption spectroscopy following standard procedures. The study protocol conformed to the ethical guidelines of the 1975 Declaration of Helsinki as reflected in a priori approval by the appropriate institutional review committee. Informed consent for liver biospsy was obtained from all patients. Additional permission to measure the human liver samples by LA-ICP-MS was given by the ethics commission located at the Medical Faculty of the RWTH University Hospital Aachen (EK256/12 and EK186/15).

**Table 1:** Patient's characteristics

Cohort	Diagnosis	*HFE* Genetics*	Sex	Age	Liver tissue Fe [μg/g]	Serum Fe [μM]	Transferrin [mg/dL]	Ferritin [μg/L]	Tranferrin saturation [%]	Anonymised Sample no.
Iron overload	*HFE* HH	Y282/Y282 H63/H63	M	61	1117	ND	138	1950	95	H4
	Non-*HFE* HH	C282/C282 H63/D63	M	55	2828	22.4	246	1850	36	H3
	*HFE* HH	Y282/Y282 H63/H63	F	60	7600	33.7	158	934	85	H1
	HCV	C282/C282 H63/H63	F	13	NID	21.4	246	52	35	H2
Normal iron status	HBV, LTx	ND	M	59	NID	16.9	254	52	26	C4
	Ductopenia, LTx	ND	F	69	NID	17.4	190	196	36	C1
	LTx	ND	F	53	NID	21.0	297	64	28	C3
	GvH	ND	M	36	NID	19.2	250	1431	31	C2

Abbreviations used: *F* female, *GvH* graft-versus-host, *HBV* hepatitis B virus, *HCV* hepatitis C virus, *HFE HH* hereditary hemochromatosis, *LTx* liver transplantation, *M* male, *NA* not applicable, *ND* not determined, *NID* histologically no iron deposition, *NN* not known. Normal laboratory reference ranges are: hepatic Fe [μg/g dry weight]: 200-2400 (M), 400-1600 (F); serum Fe [μM]: 9-29 (M), 9-27 (F); Transferrin [mg/dL]: 200-400; Ferritin [μg/L]: 30-300 (M), 10-200 (F); Transferrin saturation [%]: 18-45. * *HFE* genotype constellations of patients subjected to LA-ICP-MS analysis were determined using TaqMan allelic discrimination assay [3, 9]

### Genetic analysis

**Fig. 1:**
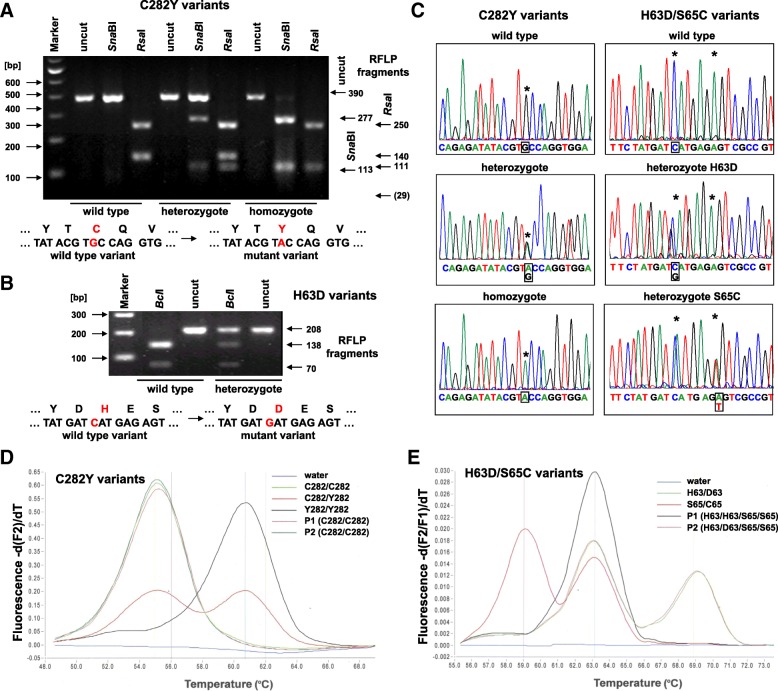
Molecular diagnosis of *HFE* gene mutations. **a** Historically the first molecular tests for detections *HFE* gene mutations were based on RFLP analysis. In a typical setting, individual parts of the *HFE* gene locus were amplified by PCR. For detection of the C282Y mutation, the amplified products were then digested with *Sna*BI (TAC↓GTA) or *Rsa*I (GT↓AC) and the resulting fragments were resolved by agarose gel electrophoresis. Heterozygote or homozygote carriers of the *HFE* C282Y are distinguished by their restriction fragment pattern following protocols described elsewhere [13]. **b** Similarly, the H63D mutation can be identified by the presence of a second *Bcl*I (T↓GATCA) restriction site that is not present in the amplicon of individuals carrying the wild type variants. **c** Sequence analysis of the C282Y and the H63D/S65C variants. **d, e** LightCycler analysis of different C282Y and H63D/S65C control probes and patient samples (P1, P2) using melting curve profiling using established protocols [10–12]. Details about *HFE* LightCycler analytics are given in Materials and Methods section

Genomic DNA was isolated from EDTA-anticoagulated whole blood. *HFE* genotyping for the p.Cys282Tyr and p.His63Asp polymorphism of patients subjected to LA-ICP-MS analysis was carried out using a validated TaqMan allelic discrimination assay using previously described protocols before [3, 9]. Lightcycler testing for *HFE* gene mutations depicted in Fig. 1 was done following established protocols [10–12]. Control DNA samples for the C282Y variant site were taken from the respective kit system (Roche Diagnostics, Mannheim, Germany). In addition, a DNA sample from a patient carrying heterozygous S65C and H63D mutations were kindly provided by Genes4U AG (Neftenbach, Switzerland). Restriction fragment length polymorphism analysis with *Rsa*I (Roche) and *Sna*BI restriction nucleases (New England Biolabs, Frankfurt am Main, Germany) for the C282Y variant and *Bcl*I restriction nuclease (Roche) for the H63D variant was done following protocols reported elsewhere [13]. Sequencing of amplified products was done with primers spanning the variant site following standard procedures.

### Iron stain in liver tissue

Ferrous ion (Fe^2+^) was determined in paraffin-embedded samples following a standard Turnbull’s blue staining procedures. In brief, tissue slides were first deparaffinized with xylene and rehydrated through a graded alcohol series. For reduction of ferric ion (Fe^3+^) to ferrous (Fe^2+^), the sections were first incubated in ammonium sulfide for 1 h and subsequently rinsed in distilled water. Subsequently, the slices were incubated in a 1:1 mixture of 20% potassium ferrocyanide and 1% HCl solution for 10 min. Subsequently, the slices were extensively washed in distilled water and counterstained in Nuclear Fast Red solution (0.1% *w*/*v* with 5% aluminum sulfate).

### Sample preparation for LA-ICP-MS measurements

The liver samples were cryo-cut into 30 μm thick slices with a CM3050S cryomicrotome (Leica Biosystems, Wetzlar, Germany) on -18 °C cryo-chamber temperature and -16 °C object area temperature, and thaw-mounted onto adhesive StarFrost® microscope slides (Knittel Glass, Braunschweig, Germany). Samples were dried and stored at room temperature.

### LA-ICP-MS set up and measurements

Prior measurement, the mounted tissues were photographed using a Nikon Eclipse E80i research microscope (Nikon, Tokyo, Japan). The LA-ICP-MS measurements for elemental Bioimaging were performed in an experimental setup in which a high performing quadrupole Agilent 7900 ICP-MS (Agilent Technologies, Santa Clara, CA, USA) was combined with a laser ablating device, allowing the sample material to be ablated line by line with a focused laser beam (New Wave UP213, New Wave Research, Fremont, CA, USA). To keep the measurement time of the mass spectrometer as low as possible and for receiving highest spatial resolution during measurement, only isotopes of interests were selected. Standards for determination of element concentrations were produced from homogenized tissue spiked with varying concentrations of a standard salt solution. More details about the precise experimental setup used during the measurements, calibration and standard preparation are given elsewhere [14–16]. The precise parameters used in LA-ICP-MS measurement are summarized in Additional file 1: Table S1.

### Image generation of bio-metal distribution

Isotope images were generated in Microsoft Excel with the help of the visualization tool ELAI (Excel Laser Ablation Imaging) as described elsewhere [16]. This operating system allows the generation of images from mass spectrometry data without the need of any further additional software. This software for easy customizable semi-manual image generation, including documentation is available free of charge and can be downloaded elsewhere [16].

## Results

**Fig. 2:**
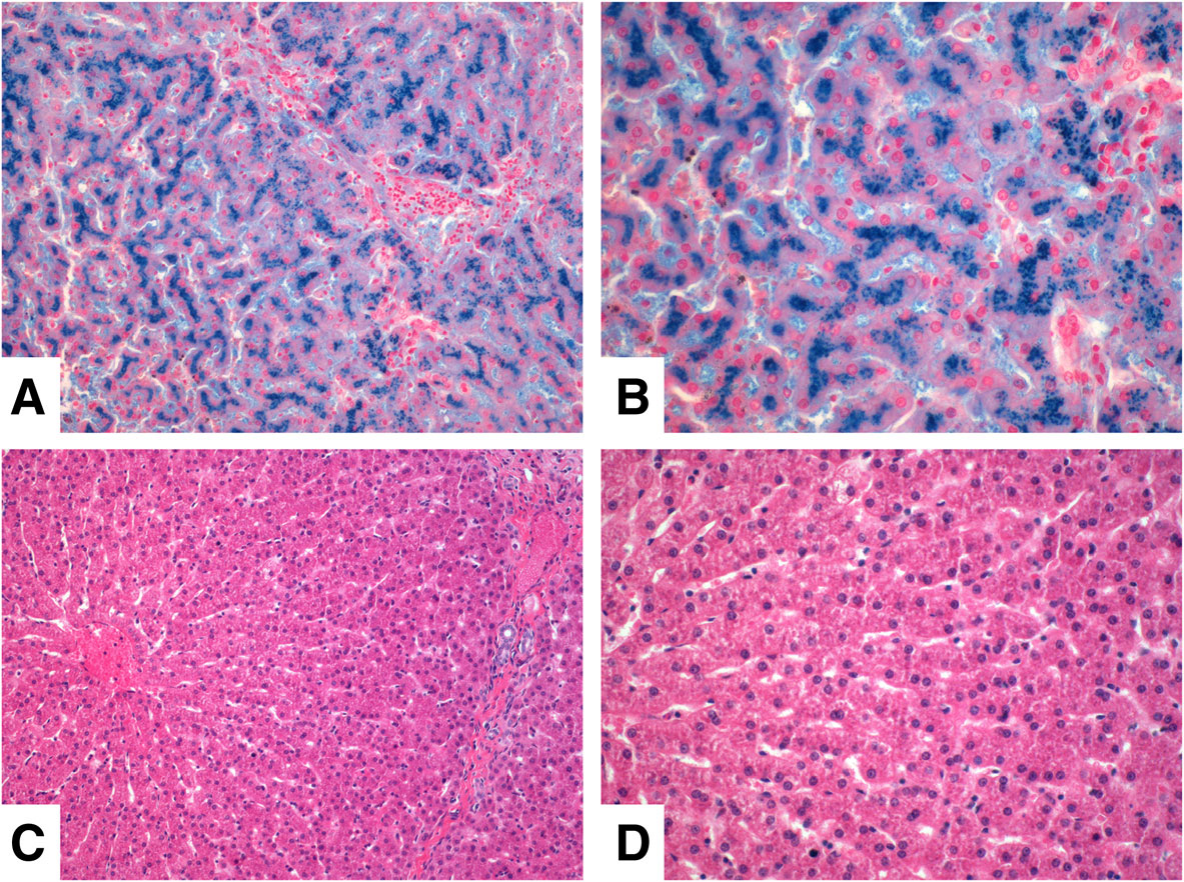
Turnbull’s blue stains and quantitative trace metal imaging in liver specimen by LA-ICP-MS. **a, b** Tumbull’s blue stain of a liver specimen obtained from a patient suffering from hereditary hemochromatosis. The entire lobule has accumulated iron and the liver is at high risk of developing significant fibrosis. Magnifications are 100x in (**a**) and 200x in (**b**). For orientations, hematoxylin and eosin (H & E) stains of normal subjects at same magnifications are shown in (**c**) and (**d**)

During the last years, we established a large variety of molecular techniques for *HFE* genotyping including restriction fragment length polymorphisms (RFLP) analysis (Fig. 1a, b), genomic sequencing (Fig. 1c), and real-time polymerase chain reaction (PCR) analytics using fluorescence resonance energy transfer (FRET) probes (Fig. 1d, e). In most cases, these genetic tests are useful in confirming the diagnosis of HH, especially in patients with clinical signs of HH or in patients with excessive accumulation of hepatic iron (Fig. 2).

**Fig. 3:**
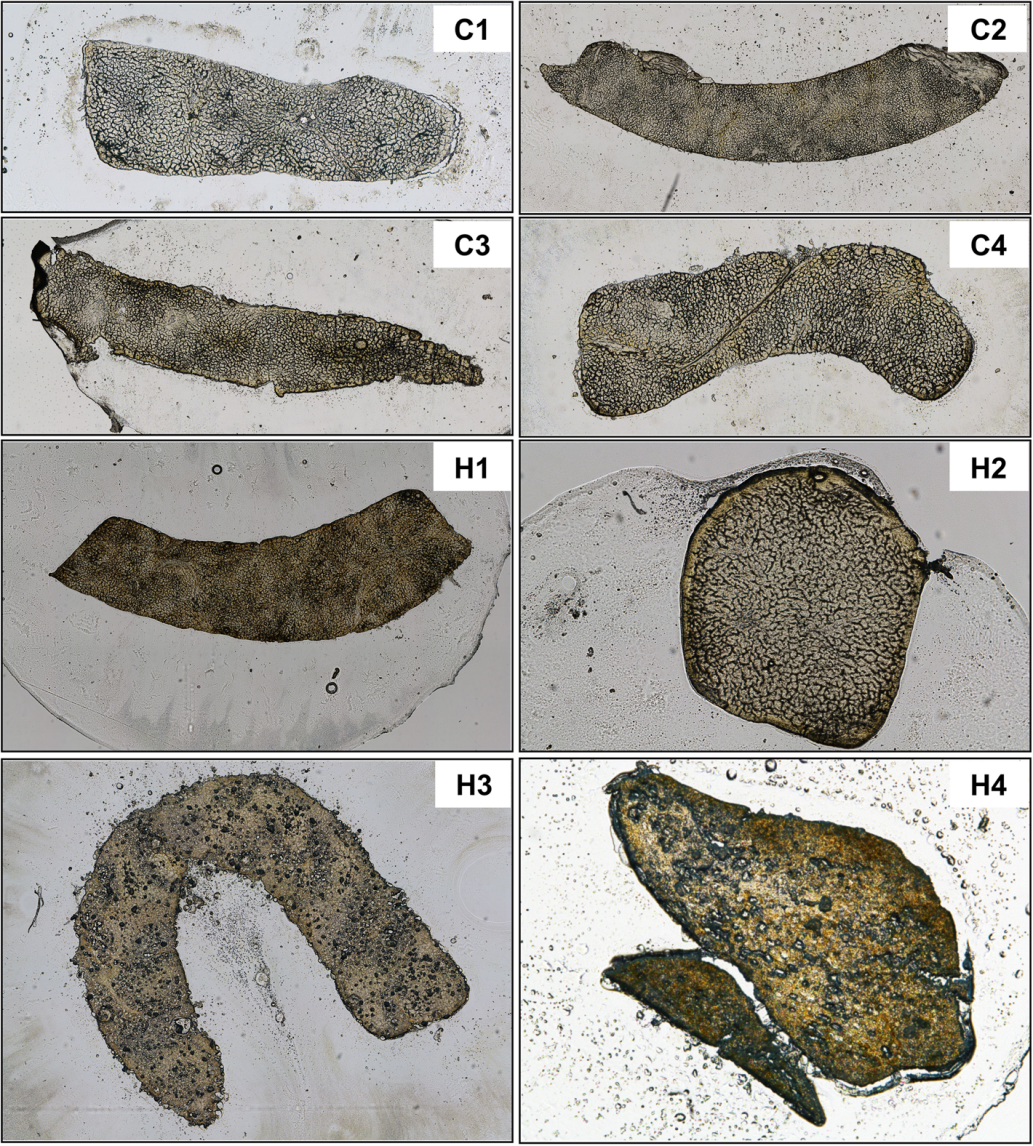
Human liver samples analyzed. Control liver samples (C1-C4) and liver samples taken from confirmed iron overload patients (H1-H4) were collected by percutaneous liver biopsy. Representative cyrosections generated thereof for LA-ICP-MS metal bio-imaging are depicted

Here we used the LA-ICP-MS technology to measure element concentrations in livers of 2 patients with *HFE*-associated HH (H1, H4) one patient with non-*HFE* hemochromatosis (H3), a non-p.Cys.282Tyr hemochromatosis patient (H2) without evidence of iron overload as assessed by analysis of representative liver biopsy specimen of respective subject, and a patient with transfusional-induced iron overload after bone marrow transplantation (C2). In addition, liver specimens taken from liver transplant recipients without biochemical evidence of iron overload were taken as controls (C1, C3, and C4). The overall morphology of respective biopsy samples is depicted in Fig. 3.

**Fig. 4:**
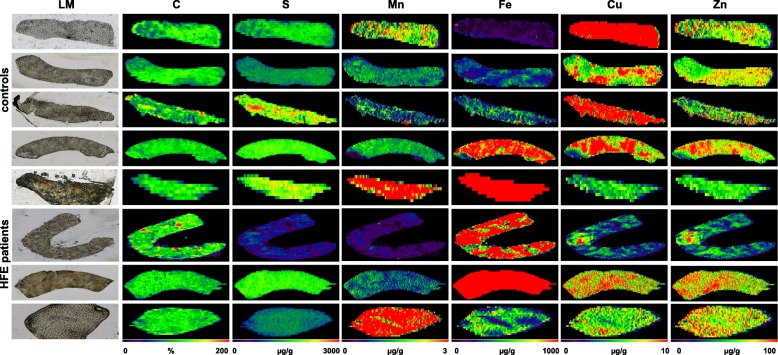
Metal imaging in liver specimen. Liver specimen with a thickness of 30 μm from each four *HFE* patients and healthy control subjects were analysed for content of carbon (C), sulfur (S), manganese (Mn), iron (Fe), copper (Cu), and zinc (Zn) by LA-ICP-MS. Individual images of elements were generated with the ELAI software tool. Light microscopic (LM) images of cryosections are depicted for orientation in the left margin (magnification × 4). The content of C used for normalization is given in %, while all other concentrations are given in μg/g liver tissue. More information about sample preparation, measurement, and data analysis are given in the Materials and Methods section. The precise parameters used in LA-ICP-MS measurement are summarized in Additional file 1: Table S1

As expected, the overall concentration of iron was significant higher in samples taken from iron overload patients. While the iron concentration in three respective liver specimens were 18,992 ± 8093 μg/g (H1), 2051 ± 1490 μg/g (H3), and 8261 ± 3405 μg/g (H4), the normal controls had overall lower concentrations (132.3 ± 126.0 μg/g (C1), 500 ± 299 μg/g (C3), and 580 ± 209 μg/g (C4)) (Additional file 2: Table S2). Only the non-p.Cys282Tyr hemochromatosis patient (H2) had somewhat lower concentration (621 ± 303 μg/g), while one bone marrow-transplanted patient with transfusional iron overload (C2) had elevated quantities (1534 ± 535 μg/g). Elevated iron concentrations in iron overload patients were also well-illustrated when we depicted the results as heat maps using the ELAI software (Fig. 4). Interestingly, the generated images revealed lower concentration of copper in *HFE* patients and large variations in manganese compared to the controls, while the concentration of zinc was comparable to those measured in healthy subjects.

**Fig. 5:**
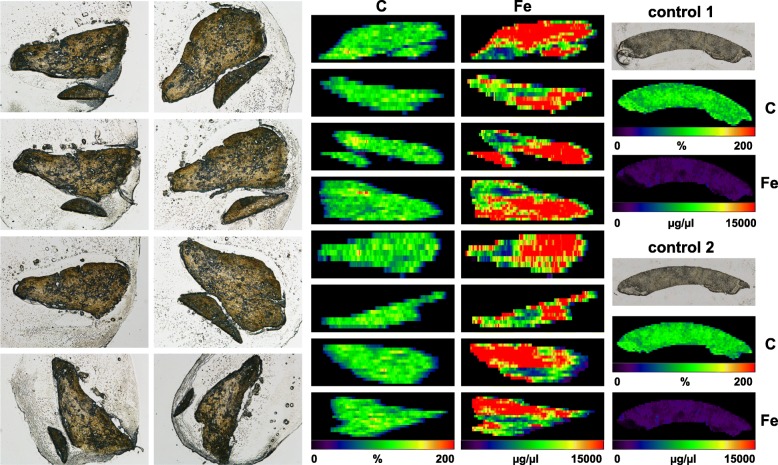
Reproducibility of LA-ICP-MS measurements. Serial sections from a liver specimen taken from a patient suffering from *HFE* liver samples (H4) were generated and analysed by LA-ICP-MS for content of carbon (C) and iron (Fe). The content of C used for normalization is given in %, while Fe concentrations are given in μg/g liver tissue. As a control, two sections from a subject (C2) with lower iron values were analyzed in parallel

However, since our measurements were only conducted in small groups, we actually do not know if these findings are of general validity or specific for the analyzed iron overload sample set (*n* = 4). We next tested the reproducibility of our measurements. Therefore, we measured the iron concentrations in eight serial cuts of the same *HFE* liver specimen (H4). In all eight cases, the determined iron concentration was visually the same showing the typical increased and uneven distribution of iron within the iron-overloaded tissue compared to samples collected from a healthy proband (Fig. 5).

**Fig. 6:**
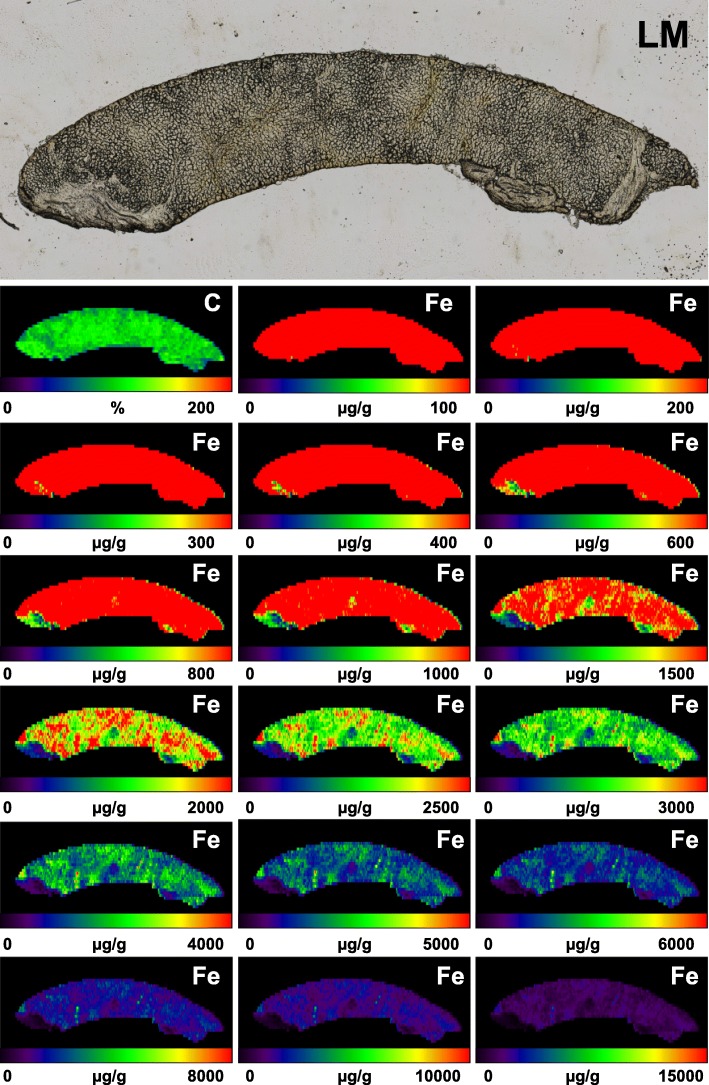
Usage of different concentration ranges for ELAI image visualization. A liver specimen (taken from patient C2) was imaged by LA-ICP-MS for carbon (C) and iron (Fe). The different images for Fe were generated by using of different concentration ranges. Most favorable for image representation are ranges in which the mean concentration of the measured element is in the middle (i.e. in this case the green range) of the scale. A light microscopic (LM) image is given for orientation

To allow best visualization of measured metal concentrations in the experimental setup, it is optimal to set the ELAI scale during data processing into a range encompassing concentrations in which the estimated mean regional concentrations are centered in the middle of the selected scale. Exemplarily, when evaluating a measurement of a healthy subject with an estimated liver iron concentration lower than the threshold 60 μmol/g (~ 3350.7 μg/g) dry liver tissue [17], a scale ranging from 0 to 2500 μg/g to 0–6000 μg/g tissue is recommended for reconstruction of metal distribution maps (Fig. 6).

## Discussion

Previously, we have optimized laser ablation inductively coupled mass spectrometry (LA-ICP-MS)-based methods for trace metal imaging in liver sections [14]. These protocols have multi-element capability suitable to simultaneously measure and quantify a large variety of different metals and metalloids within the tissue with high spatial resolution. In brief, a focused laser beam ablates a small quantity of biopsy material, and the aerosol produced is transported in an inert carrier gas stream to an ICP-MS, where it is then atomized and ionized. Subsequently, the different ions are separated according to their mass-to-charge ratio and quantified [15].

As demonstrated in our study, the LA-ICP-MS technique allows precise measurement and visualization of iron concentrations in high resolution. This is a great advantage when compared to other standard techniques. Quantitative measurements by spectrophotometry or semi-quantitative iron determinations by histology suffer from two limitations. The within-organ standard deviation (SD) of hepatic iron concentrations (HIC) can vary widely. Moreover, HIC values determined in microtome samples and biopsy-sized samples can have large coefficients of variations (CVs) reaching values of up to 71% in patients with end-stage cirrhosis [18]. It is also reasonable to speculate that histological stains are only semi-quantitatively because these are artificially lowered by removal of iron from the liver tissue during the fixing, washing and staining steps in the histochemical procedure. Likewise, the chemical determination of total iron in percutaneously obtained liver biopsy from patients with suspected primary iron overload identified by colorimetric analysis, flame atomic absorption and flameless atomic absorption spectrophotometry by a graphite furnace method revealed CVs ranging from 11 to 19% resulting from sample variation due to inhomogeneous distribution of iron through the liver [19]. Moreover, quantification of liver iron with MRI, computed tomography (CT), magnetic resonance spectroscopy (MRS), liver susceptometry, and relaxometry are partially limited. Although they are rapid, non-invasive, and cost effective techniques limiting the use of liver biopsy in assessment of liver iron content, these methods have some analytical drawbacks [20, 21]. In particular, the occurrence of concomitant fat, inflammation and fibrosis within the liver corrupts the ability of gradient echo methods, often requiring the correlation with chemically determined liver iron concentration for establishment of empirical calibration curves. This provides a challenge in current MRI measurements of precise liver iron concentration in correcting for the transverse rate R2 (= 1/T_2_) and the faster and more sensitive R2* (= 1/T_2_*) cellular interference, including fibrosis, fat, inflammation, and other histologic changes in hepatic cellularity that are associated with tissue damage resulting from iron overload [22].

In addition, iron-overloaded livers can show iron heterogeneity over spatial scales spanning three orders of magnitude in regard to intracellular, intercellular and zonal compartments. These circumstances affect relaxation times during measurements, thereby introducing unavoidable errors [20].

In the last decade, several efforts were made to overcome these potential limitations in MRI and CT. Exemplarily, a new R2-MRI imaging technology termed “FerriScan” was introduced some years ago. This technology allows accurate measurement of liver iron concentration with high sensitivity and specificity and is unaffected by hepatic fat content, inflammation, fibrosis or cirrhosis [23, 24].

LA-ICP-MS imaging is a sophisticated tool for investigating the regional and spatial distribution of metals with high sensitivity, capability, and relatively good lateral resolution at micrometer resolution [25]. Therefore, this technology is becoming an essential tool in diverse biological research fields, and of course in clinical applications. We here have demonstrated that LA-ICP-MS is highly suitable to measure and localize iron concentrations of deposits in liver samples. Therefore, this technique might be relevant in the more precise determination of hepatic iron status, localization of iron deposits, or in HIC monitoring during HH therapy.

Presently, therapeutic phlebotomy is the standard clinical practice in the therapy of HH [26]. However, there is no evidence base on which to direct the optimal endpoint of this therapy. Actually, most clinicians attempt to achieve a target of serum ferritin lesser than 50 μg/L. However, this value does not necessarily correlate with hepatic iron content. Possibly, the direct measurement of iron concentration and distribution in liver biopsy with LA-ICP-MS will be more suitable to mark a therapeutic endpoint in the treatment of HH patients.

The LA-ICP-MS protocols used in our study will be potentially relevant not only in estimating the degree of iron overload in hemochromatosis patient. There is a large set of other genetic or acquired disorders that lead to strong imbalances in iron homeostasis. Beside hemochromatosis, system iron overload syndromes can have many other genetic or acquired origins including hereditary aceruloplasminemia, dyserythropoiesis, different forms of β-thalassemia, and several other conditions requiring multiple transfusions including myelodysplasia or hematopoietic stem cell transplantation resulting in iatrogenic iron overload [27–29]. Likewise, monosyndromic or polysyndromic sideroblastic anemias are known to develop both compartimental iron excess and systemic iron overload [27]. On the other side, iron malabsorption, internal chronic gastrointestinal bleeding, excessive menstrual bleeding, pregnancy, parasitic infection, and nutritional deficiencies resulting for example from strict vegetarian food consumption can provoke iron shortcomings in body’s iron homeostasis [30, 31] that might be also reflected in lowered liver iron content.

## Conclusions

**Fig. 7:**
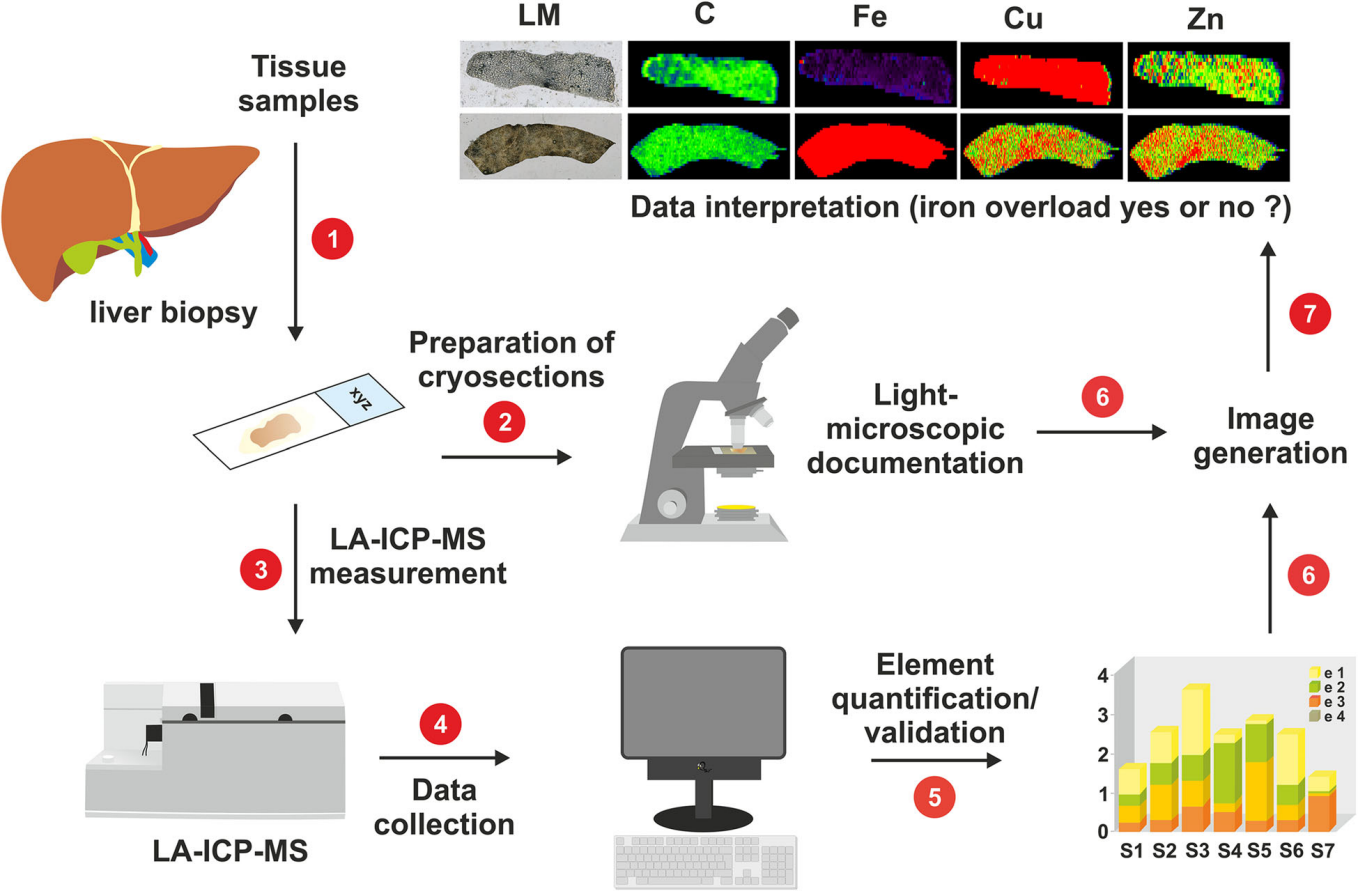
Simplified scheme of LA-ICP-MS workflow in imaging of iron overload. Metal imaging in the liver biopsy specimen requires seven steps including (1) the preparation of the cryosection with a precisely defined thickness (30 μm), (2) the light microscopic documentation of the section, (3) the LA-ICP-MS measurement itself, (4) data collection and compilation, (5) computation and validation of absolute or relative element concentration, (6) creation of colour-coded images of measured elements, and finally (7) data interpretation and making the concluding diagnosis “iron overload” or “no iron overload”, respectively

In conclusion, our study aimed to improve the performance of LA-ICP-MS for routine measurement in HH diagnosis and other iron-related dysbalances. The respective workflow containing seven individual steps (Fig. 7) is straightforward and creates images that are easy to interpret. The data presented suggests LA-ICP-MS biometal imaging is a significant asset in the diagnostic evaluation of hereditary hemochromatosis, where the simultaneous measurement of iron and other metals offers unique opportunities for deeper understanding of the biology of hemochromatosis and iron overload.

## Additional files

Additional file 1: **Table S1.** Operating parameters of LA-ICP-MS imaging of human liver samples. (DOC 45 kb)

Additional file 2: **Table S2.** Element concentrations in analysed liver samples. (DOC 40 kb)

## Abbreviations

CV: Coefficient of variation
ELAI: Excel Laser Ablation Imaging
*HFE*: Gene encoding the hemochromatosis gene
HH: Hereditary hemochromatosis
LA-ICP-MS: Laser ablation inductively coupled plasma mass spectrometry
