# Supplementary material for: Quantification of liver iron overload disease with laser ablation inductively coupled plasma mass spectrometry
Source: BMC Med Imaging. 2018 Dec 4;18:51. doi: 10.1186/s12880-018-0291-3 (PMC6278171; doi:10.1186/s12880-018-0291-3)
Supplement: Supplementary file 1 — Table S1. Operating parameters of LA-ICP-MS imaging of human liver samples. (DOC 45 kb) [file 12880_2018_291_MOESM1_ESM.doc]

**Table S1**

Operating parameters of LA-ICP-MS imaging of human liver samples

| **Parameter** | **Setting** |
| --- | --- |
| Rf power input | 1450 W |
| Cooling gas flow rate | 16.0 L min^-1^ |
| Auxiliary gas flow rate | 0.7 L min^-1^ |
| Carrier gas flow rate | 1.0 L min^-1^ |
| Dwell time | 20 ms |
| Extraction lens potential | 3400 V |
| Mass resolution (m/Δm) | 300 |
| Scanning mode | peak hopping |
| Analysis time per liver sample*  (10 mm × 10 mm) | 4 - 6 hours |
| Laser ablation system | New Wave (UP213), New Wave Research Inc., Fremont, CA, USA |
| Wavelength of Nd:YAG laser | 213 nm (deep UV) |
| ICP-MS | Agilent 7900, Agilent Technologies Inc., Santa Clara, CA, USA |
| Laser fluence | 0.24 J cm^-2^ |
| Repetition frequency | 20 Hz |
| Laser spot size | 60 µm |
| Scan speed* | 50- 60 µm s^-1^ |
| Ablation mode | line scan |
| Isotopes monitored | C^13^, Na^23^, Mg^24^, Mg^25^, P^31^, S^33^, S^34^, K^39^, Ca^43^, Ca^44^, Cr^52^, Mn^55^, Fe^56^, Fe^57^, Ni^60^, Cu^63^, Zn^64^, Cu^65^, Zn^66^, Br^79^, Br^81^, Pb^208^ |

**Please note**: * Scan speed was selected in dependence on cryosample size.
