# Supplementary material for: Quantification of liver iron overload disease with laser ablation inductively coupled plasma mass spectrometry
Source: BMC Med Imaging. 2018 Dec 4;18:51. doi: 10.1186/s12880-018-0291-3 (PMC6278171; doi:10.1186/s12880-018-0291-3)
Supplement: Supplementary file 2 — Table S2. Element concentrations in analysed liver samples. (DOC 40 kb) [file 12880_2018_291_MOESM2_ESM.doc]

**Table S2**

**Element concentrations in analysed liver samples**

|  | **Controls** | | | | **Iron overload patients** | | | |
| --- | --- | --- | --- | --- | --- | --- | --- | --- |
| **Isotope** | **C1***  **µg/g**  **(Mean ± SD***)** | **C2**  **µg/g**  **(Mean ± SD)** | **C3**  **µg/g**  **(Mean ± SD)** | **C4**  **µg/g**  **(Mean ± SD)** | **H1**  **µg/g**  **(Mean ± SD)** | **H2**  **µg/g**  **(Mean ± SD)** | **H3**  **µg/g**  **(Mean ± SD)** | **H4**  **µg/g**  **(Mean ± SD)** |
| **^13^C**** | 100 ± 22.4 | 100 ± 13.3 | 100 ± 30.4 | 100 ± 19.5 | 100 ± 17.3 | 100 ± 17.7 | 100 ± 35.9 | 100 ± 20.7 |
| **^34^S** | 1199 ± 253 | 1774 ± 214 | 2028 ± 501 | 2128 ± 307 | 1718 ± 227 | 1089 ± 163 | 541 ± 240 | 1023 ± 187 |
| **^55^Mn** | 1.65 ± 0.875 | 1.225 ± 0.419 | 1.084 ± 2.40 | 5.51 ± 1.99 | 1.002 ± 0.376 | 3.81 ± 1.57 | 0.353 ± 0.198 | 0.648 ± 0.230 |
| **^56^Fe** | 132.3 ± 126.0 | 1534 ± 535 | 500 ± 299 | 580 ± 209 | 18992 ± 8093 | 621 ± 303 | 2051 ± 1490 | 8261 ± 3405 |
| **^63^Cu** | 43.8 ± 29.3 | 9.3 ± 5.59 | 20.1 ± 16.4 | 8.0 ± 3.14 | 7.97 ± 2.99 | 5.96 ± 2.34 | 3.13 ± 1.95 | 3.74 ± 1.14 |
| **^64^Zn** | 89.1 ± 44.2 | 111.8 ± 33.3 | 89.4 ± 56.3 | 128.3 ± 39.0 | 116.8 ± 38.9 | 110.8 ± 36.5 | 54.1 ± 29.3 | 57.5 ± 14.5 |

**Please note**: * for overall morphology of samples C1-C4 and H1-H4 see Figure 3. ** Element concentrations were normalized to the content of carbon (^13^C). *** A high standard deviation (SD) indicates that the measured element is not uniformly distributed in the liver section.
